# Health Tract No. 352

**Published:** 1868-11

**Authors:** 


HEALTH TRACT NO. 352.

### BED-ROOMS.

As one-third of our entire existence is spent in our chambers, in the unconscious happiness of sleep, and as good health is impossible without the habitual breathing of a healthy atmosphere, the importance of inhaling a pure air during sleep is self-evident. No sleep can be sound and healthful, unless the person is comfortably warm; and many a man who has gone to bed in health has awakened with a mortal malady, or one involving life-long suffering, by having been exposed to a draft of air upon some part of the body while asleep, either from an open door, an open window, a broken pane or an unstopped crevice.

Three things then are indispensable to the healthfulness of a bed-chamber; we must be comfortably warm, must not be exposed to drafts of air, and must be supplied with a pure air, not very cold. A great deal has been written about sleeping with windows sky-high, so as to let in all out of doors; none but monomaniacs or born fools write thus; we know that many persons have met their deaths by having been exposed by means of an open window to a sudden change in the weather during the night; and certainly the safe side is the best.

In cold weather there should be a fire in an open fire-place all night, and air enough will get in at the crevices of the doors and windows to create a current, driving the bad air up the chimney. In summer, a lamp or candle may be burned standing in the fire-place, unless the door of the hall is left open; but as most persons, at least in cities, do not feel safe to sleep with an open door, the lamp is a good substitute. A window may be hoisted, but there are comparatively so few nights during the year to make it safe to do so, that the fire or open inner door is preferable. There is no advantage in going to bed or undressing in a cold room; all invalids and sedentary persons should undress, sleep and arise, in a room not lower than fifty degrees; and if it was seventy, while rising, so much the better. The old, the sedentary and the sickly, should sleep on feather-beds in cold weather; if they sleep on matrasses, it often requires so much bed clothing to keep them comfortably warm, that it oppresses the breathing and so confines the foul air above the bed, as to make them restless.

**In a close room, the first out breathing contaminates the whole volume of air in the apartment; and this will go on until at last there is not enough pure air to sustain life, and the man dies; but when the hot air comes in at the ceiling, it forces the bad air to the floor, so that if holes are open in the floor, or around the base-board, and the floor is furred, this bad air escapes in that direction, heating the floor in its passage outwards, thus effectually preventing cold floors, which cause cold feet, making our wives and daughters cross and enriching the Doctor and the apothecary.**

